# Isolation of *Raoultella ornithinolytica* in a HIV positive patient with a lung abscess

**DOI:** 10.1099/acmi.0.000365

**Published:** 2022-06-08

**Authors:** Nicola Hinchcliffe, Aled Benbow, Tara Moshiri, Jonathan Thompson, Pradhib Venkatesan

**Affiliations:** ^1^​ Department of Infectious Diseases, Nottingham University Hospitals, City Campus, Nottingham, NG5 1PB, UK

**Keywords:** *Raoultella*, *R. ornithinolytica*, lung abscess, HIV, I6s rRNA

## Abstract

A 38 year old male HIV positive patient with a history of intravenous drug use presented with chest pains, cough, sputum and weight loss and radiology demonstrated the evolution of a right basal lung abscess. A lung biopsy sent for 16S rRNA analysis and sputum cultured about the same time demonstrated *Raoultella ornithinolytica *. No other causative pathogens were clearly identified. He gradually improved with a 4 week course of intravenous cefazolin. *R. ornithinolytica* is a rare, but recognised pathogen.

## Introduction

*Raoultella* species are part of the *Enterobacteriaceae* family and through genomic analysis have been separated from *Klebsiella* species [1]. There are four main species: *Raoultella planticola *, *Raoultella ornithinolytica *, *Raoultella terrigena* and *Raoultella electrica* [2]. *Raoultella* species have been found in the environment in water, soil and plants, but also noted as colonising the respiratory and gastrointestinal tracts of humans. Infections have been most often associated with *R. planticola* and less often with *R. ornithinolytica* [2].

Series of *R. ornithinolytica* isolates have been described from South Korea, Portugal and France. Over a 10 year period a tertiary care centre in Seoul, South Korea with 1983 beds identified 16 cases from blood cultures [3]. Over a 5 year period a tertiary centre in Lisbon, Portugal, isolated *R. ornithinolytica* on 25 occasions [4]. Between 2002 and 2013 four hospitals in Southern France with a total of 4000 beds recorded 225 isolates of *R. orinithinolytica* [5]. Thus *R. ornithinolytica* infections are not common, but rates of identification may be underestimated as phenotypic methods may misclassify isolates [2, 5]. When mixed cultures are reported it is not clear whether *R. ornithinolytica* is a pathogenic contributor. We report a HIV patient with a lung abscess whose only positive microbiological finding was *R. ornithinolytica *.

## Case report

A 38 year old man presented to hospital with a 2 week history of being unwell. His background history included intravenous drug use, HIV, untreated hepatitis C, seizures and an allergic rash to both penicillin and co-trimoxazole. He smoked up to 20 cigarettes per day and also smoked heroin. He reported no longer being able to access his veins to administer intravenous drugs. Regarding his HIV he only attended clinic sporadically and was not adherent to anti-retroviral medication. For 2 years prior to admission his CD4 count was consistently below 50×10^6^ cells per litre. One year before this presentation he had been treated for *Pneumocystis jirovecii* pneumonia (PCP). He presented with pleuritic chest pains, cough with some green to brown sputum but without haemoptysis, shortness of breath, night sweats, extreme lethargy and reported a 12 kilogram weight loss over 1 month. He had poor dentition and his BMI was 18. He was afebrile with a respiratory rate of 28 per minute and oxygen saturations were 95 % on room air. He had bi-basal pulmonary crackles. He had very dry skin and scattered skin excoriations.

### Investigations

**Fig. 1.**
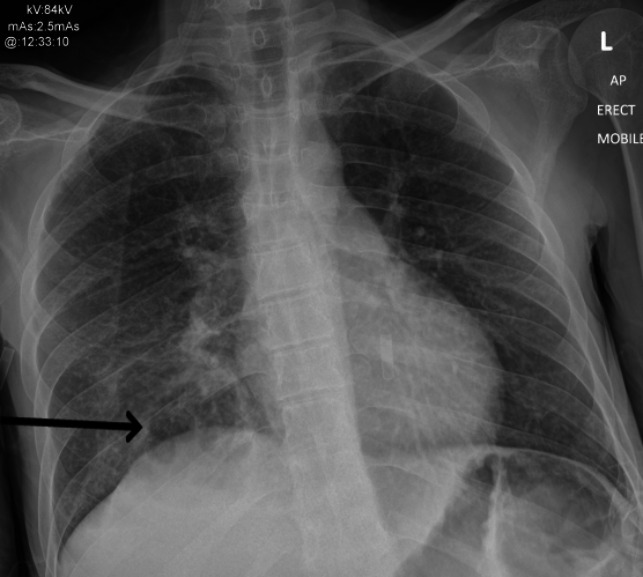
Chest X ray on admission. Early right lower zone cavitation is indicated by the arrow.

The admission chest x-ray showed early right lower zone cavitation (Fig. 1). A SARS-CoV-2 nasopharyngeal swab was negative. On admission he was commenced on second line empirical treatment for PCP in the form of clindamycin plus primaquine and was also given doxycycline, all orally. Over the following days further results showed that sputum culture only contained respiratory commensals, three sputa were acid fast bacilli (AFB) smear negative (and later also culture negative), a throat swab and sputum were *P. jirovecii* PCR negative, urinary *Legionella* and pneumococcal antigens were not detected and both *Aspergillus* galactomannan and beta-d-glucan were negative. His HIV viral load was 605085 copies per millilitre and his CD4 count was 5×10^6^ cells per litre.

Venous access was difficult and a peripheral venous catheter was inserted radiologically. In total he had four sets of blood cultures. His first blood culture was taken from a forearm vein and later cultures through the newly inserted venous catheter. His first blood culture grew *Staphylococcus aureus* in one bottle, which proved resistant to clindamycin, but sensitive to flucloxacillin, gentamicin, doxycycline and ciprofloxacin. A total of seven blood culture bottles proved culture negative.

### Diagnosis

**Fig. 2.**
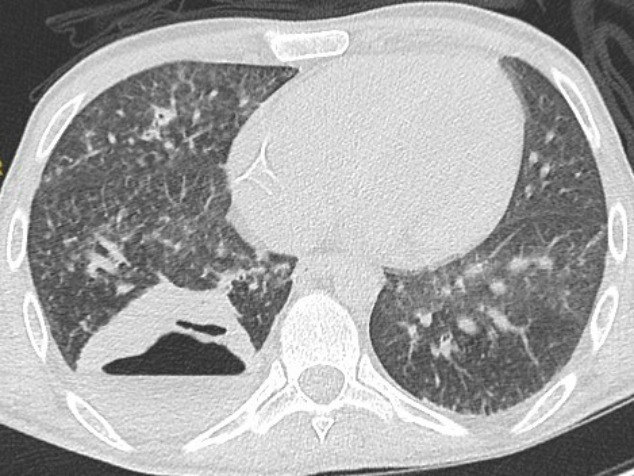
CT scan of chest 7 days post-admission. A 73×69 mm thick walled abscess cavity with a fluid level is seen in the right lung.

The patient’s condition began to deteriorate 5 days into his admission with fever, hypoxia and an increasing oxygen requirement. His antibiotic treatment was converted to intravenous cefazolin 2 grams three times a day. A CT chest scan was performed, which revealed a 73×69 mm thick walled abscess cavity in the right lower lobe and two other smaller cavities (Fig. 2). Four 18 g cores were taken from the largest abscess under CT guidance. Initial, extended cultures (bacterial, AFB and fungal) did not yield any growth and histological analysis was in keeping with abscess formation. Lack of growth on culture could relate to suppression from the administered antibiotics or low bacterial load in the biopsy sample.

The biopsy sample was sent to an external, commercial laboratory for 16S rRNA sequencing which returned the closest matches as *Raoultella* species and *Klebsiella aerogenes *. The assay involves sequencing a conserved region of the 16S rRNA gene and comparing this to the GenBank database. This result was correlated with a sputum sample obtained the day before the biopsy. The sputum grew *Raoultella ornithinolytica* on Columbia Blood Agar and Chocolate Agar with 7 % Defibrinated Horse Blood and Bacitracin. Identification was by matrix-assisted laser desorption/ionisation – time of flight mass spectrometry (MALDI-TOF). Antibiotic susceptibilities were established by a commercial broth microdilution assay using the MicroScan system. This found resistance to amoxicillin and fosfomycin and sensitivity to all other targets which included: co-amoxiclav, ceftazidime, cefotaxime, piperacillin-tazobactam, meropenem and other non-beta-lactam based antibiotics.

### Treatment

He began to improve on cefazolin and had a course of 4 weeks. The duration was guided by resolution of symptoms, normalisation of inflammatory markers, and reduction in the size of the abscess demonstrated on plain film radiography.

## Discussion

Infections caused by *R. ornithinolytica* have been reported from different sites including bacteraemias, urinary tract infections, including catheter associated infections, respiratory, gastrointestinal, biliary, skin/soft tissue, bone/joint infections and central nervous system infections [5, 6]. Whilst infections may occur in the previously healthy [7–9], many are reported in patients with co-morbidities, and some after surgical procedures [10, 11]. Most reported bacteraemias are linked to malignancy in various age groups [12, 13], and some from children [14–16]. Our patient had a lung abscess. Previously reported respiratory infections include pneumonia and pleural effusions [5]. Reported pneumonias were mostly associated with a chronic condition such as diabetes, alcoholic liver cirrhosis, malignancy, solid organ transplant or ventilator associated [17]. We have not identified a previous report in a patient with HIV infection.

There is always doubt if a microbiological identification signifies the true causative pathogen. Our patient had poor dentition and may have aspirated. In this context lung abscesses may be polymicrobial. However, 16S rRNA analysis implied *Raoultella* species in the lung biopsy sample. A lung biopsy was taken after the patient commenced antibiotics and this would have compromised culture results. However, one would not expect a similar effect on 16S rRNA analysis. Furthermore, *R. ornithinolytica* was found in sputum and thus present in two samples. If it had only been found in sputum one might have interpreted this as contamination from oral colonisation. *Staphylococcus aureus* was isolated from a single blood culture bottle but was not found on repeated blood cultures, nor was it found in any other cultured sites. The isolate was potentially a skin contaminant. He did have dry skin and skin excoriations. He did not improve when on oral doxycycline.

The development of a lung abscess implies pathogen and/or host factors in tissue destruction, with cell death and breakdown of extracellular tissue matrix. Whilst inflammatory neutrophils and macrophages can release matrix metalloproteases the question is whether pathogens can contribute proteolytic enzymes and factors capable of inducing cell death. In the case of *Klebsiella *, necrotising pneumonia is described. Live, encapsulated *Klebsiella pneumoniae* induces airway epithelial cell death via a mechanism involving its capsular polysaccharide [18, 19]. Like *Klebsiella *, *R. ornithinolytica* is encapsulated. To date cell cytotoxicity has not been described via capsular polysaccharide but via peptides and glycolipids. A low molecular weight fraction of supernatants from *R. ornithinolytica* cultures is cytotoxic to HeLa cells, although not to fibroblasts, through casein-derived peptides, while a high molecular weight fraction kills *Mycobacterium smegmatis* through a glycolipid-peptide complex [20, 21].

This patient responded to treatment with cefazolin. The sensitivity pattern of our isolate is consistent with the published phenotypic sensitivity profile of *R. ornitholytica* [22]. *R. ornitholytica* possesses *bla_ORN-1_* which is a chromosomally encoded class A beta-lactamase that is readily inhibited by clavulanic acid [22]. Though not apparent in this case there are multiple examples in the literature of *R. ornitholytica* acquiring additional resistance mechanisms through mobile genetic elements [23–25]. Reports include both extended-spectrum beta-lactamase and carbapenemase enzymes. The result is an organism which seems readily able to become resistant to the majority of beta-lactam based antibiotics through horizontal gene transfer. This is important to consider in settings with a high prevalence of plasmid-borne carbapenemase enzymes.

*R. ornithinolytica* infections are reported rarely and we describe the first case of its isolation from a HIV positive patient with a lung abscess.
